# News and Events of the Week

**Published:** 1910-09-17

**Authors:** 


### News and Events of the Week.

A case of "spotted fever," the first for some months past, has been admitted to the Metropolitan Asylums Board's Eastern Hospital from Bethnal Green.

Mr. William Henry Bagnell, L.R.C.P., L.R.C.S.I., of Pau, France, who died on May 6 last, aged fifty-two years, domiciled in Ireland, has left personal estate in the United Kingdom valued at £35,915.

A private conference of the official delegates of the various Governments at the Pure Food Congress at Paris, says the *Times* correspondent, has arranged to make certain methods of analysis international, with the consequence that when any food is in future submitted to an analytical test it will have to conform to that international standard.

Messrs. Newton, Chambers and Co., Ltd., of Thorncliffe, near Sheffield, have been awarded a Grand Prix by the judges at the Japan-British Exhibition for their disinfectant fluid Izal. Although premier honours have been awarded to Izal on many former occasions, this latest recognition bears fresh witness to the reliability and excellence of this well-known disinfectant preparation.

Two cases of cerebro-spinal fever have been notified at Sandon, a village about three miles from Chelmsford. One of them, which has terminated fatally, occurred about a week ago, the patient, a child, dying within forty-eight hours of the onset of the disease. This was the first case of the malady to be recognised, but an investigation resulted in the detection of the second case, which, owing to its mild character, had not previously been discovered.

The opening of the winter session and the distribution of prizes of the University College Hospital Medical School will take place on Monday, October 3, at 3 o'clock p.m., when an introductory address will be delivered by the very Rev. the Dean of Salisbury. The chair will be taken by Sir Thomas Barlow, Bart., K.C.V.O., M.D., F.R.S., President of the Royal College of Physicians. At the conclusion of the address the medical school will be open for inspection by visitors.

Messrs. Kegan Paul, Trench, Trubner and Co. announce for publication in October a selection from the remarkable essays which Mr. Arnold White, over the pseudonym "Vanoc," has contributed for two or three years past to the front page of the *Referee*. They cover a wide field of observation and reflection, from naval armaments and the ways of the War Office to "eugenics" and education, forestry and gardening, marriage and divorce. The volume will be entitled "The Views of Vanoc: An Englishman's Outlook," and will be issued at 5s. net.

The following resolution has been passed by the Committee of the Humanitarian League :—"That this Committee desires to express its satisfaction at the changes which the Home Secretary has introduced, or proposes to introduce, in the prison system—reforms which have been repeatedly advocated by humanitarians—and its hope that he will further strengthen his scheme by putting an end to all needless imprisonment for the non-payment of debts, as well as for the non-payment of fines, and by revising the system by which, under the present prison rules, a court of visiting justices, sitting in secret, can pass severe sentences of flogging on a prisoner who is undefended."

The National Bank of Spokane, Washington, announces the issuance of alleged antiseptic, germ-proof National banknotes. Fifty thousand dollars in bills recently sent out were printed with ink consisting largely of carbolic acid!

The Medical Exhibition opens at the Horticultural Hall on October 3. Full particulars may be obtained on application to the publishers of the "British and Colonial Druggist," who will forward tickets to medical men desirous of visiting the Exhibition.

The latest feature in the campaign against consumption which is being undertaken with so much vigour by the National Association for the prevention of the disease is a proposal for postal propaganda. The Bill Posters' Associations are offering their help, and 30,000 broadsides are being printed, measuring about 10 feet by 7 feet. The design, printed in eight colours, is a slight adaptation of Sir Joshua Reynolds's figure of Faith.

The following are the arrangements at the Medical Graduates' College and Polyclinic, 22 Chenies Street, W.C., for the coming week :—September 19, at 4 p.m., Dr. S. E. Dore (Skin); September 20, at 4 p.m., Dr. Theo. Thompson (Medicine); September 21, at 4 p.m., Mr. Lockhart Mummery (Surgery); September 22, at 4 p.m., Mr. R. P. Rowlands (Surgery), and September 23, at 4 p.m., Mr. Harold Barwell (Ear, Nose, and Throat).

Mr. Steet is the oldest Fellow of the Royal College of Surgeons of England. He is ninety-two years of age, and his memory and mental faculties are quite unimpaired. He became a member of the College in 1840, and took the Fellowship in 1849. He was Chief Medical Officer at the General Post Office for twenty years, and retired in 1891. He was also Consulting Medical Officer to the Spanish Consulate in London.

In one institution alone during last year upwards of 14,000 cats were received off the streets, almost all of whom were deserted and homeless, and many of them were in a horrible condition from starvation and disease. There are now upwards of forty cat institutions and shelters of one kind or another in London and the provinces, which are supported entirely by voluntary contributions. The Editor, *Animals' Friend*, wishes to remind our readers that on October 1, which is Cats' Day, they should send to the home which is nearest to them whatever they can spare. A list of the institutions can be obtained on application at York House, Portugal Street, W.C.

The Medical Officer of Hastings reports a marked decrease in the birth-rate of that borough. "As compared with the returns of previous years at the corresponding period there was in the current year a considerable decline in the number of births registered. Births of males were 125, of females 119, giving a total for the quarter of only 244 births, against 281 in 1909, 292 in 1908, and 304 in 1907. The birth-rate was thus only 14.3 per thousand of estimated population, compared with 16.5 in 1909 and 17.3 in 1908. The natural increase of the population, or excess of births over deaths in the quarter, was only 37, compared with 78 in 1909. The death-rate for the quarter was 12.1 per thousand, compared with 11.9 in 1909 for the corresponding season."

The second International Conference for the study of cancer will be held at Paris on October 1-5 next. A large number of official delegates from all parts of the world have already notified their intention of attending the Conference. Professor Czerny, of Heidelberg, will preside.

The New York Academy of Medicine has received under the will of the late Mrs. Margaret E. Gray a bequest of £10,000 to establish the Landon Carter Gray memorial, in memory of her husband. The income of the fund is to be used from time to time in purchasing books for the library of the Academy.

Dr. Jacobsohn, of Charlottenburg, has discussed the subject of Beethoven's deafness in a recent number of the *Deutsche medizinische Wochenschrift*, and thinks that it was due to a progressive otosclerosis. There is some evidence that the composer had "abdominal typhus" about a year before his hearing gave way. But little was known of typhoid at that time—it had not been differentiated from pestilential typhus. We know to-day that typhoid is a not infrequent cause of otosclerosis. There is absolutely no evidence that the composer had ever contracted syphilis, but attempts have been made to show that his skull formation was suggestive of congenital syphilis.

### THE CONGRESS OF THE ROYAL SANITARY INSTITUTE.

We have received proofs of the papers read before the Twenty-fifth Congress of the Royal Sanitary Institute, held at Brighton, September 5 to 10, 1910. The inaugural address was delivered by the President, the Hon. Sir John A. Cockburn, K.C.M.G., M.D., in which he dwelt upon the growth of sanitary science during the past few years, a growth which is epitomised in the history of the Royal Sanitary Institute. He pointed out the interest now taken in sanitary matters by municipal authorities, and showed that the trend of public opinion was towards a general recognition of the virtues of cleanliness, fresh air, pure food, and preventive medicine. As examples of the results of this last he instanced the great drop in the white death-rate on the African coast consequent on the measures introduced for the destruction of the mosquito, the gradual drop in the incidence of consumption under the influence of light and air, and the prevention of promiscuous expectoration. He called attention to the disastrous effects of syphilis both on the individual affected and on unborn generations, and pointed out the stupidity of ignoring its existence from motives of prudery, motives which denote not a virtue, but a malevolent vice which should not be permitted to be triumphant. The disease remains unnotifiable, though its effects are sapping the vitality of the nation, while other diseases of an infectious nature, which have sequelæ only on the life of the individual, are treated with commendable thoroughness. Women have taken up the study of hygiene, and their influence can only make for good and the ultimate triumph of rational eugenics. As the result of increased interest in public health, the status of the medical man has improved. The Medical Officer of Health has become as it were the High Priest of our social salvation. Lectures were delivered to the Congress on "The National Importance of Child Mortality" by Dr. Newsholme, M.D., F.R.C.P.; on "The Bricks Wherewith the Body is Built, or Man under the Microscope: a Study of his Tissues and Organs," by Dr. Alex. Hill, M.A., M.D., F.R.C.S., J.P.; on "Sanitary Science and Preventive Medicine," by Professor E. W. Hope, M.D., D.Sc.; and on "Engineering and Architecture," by Henry Rofe, M.Inst.C.E. At the meetings of the various other sections addresses were delivered by their respective presidents. That of the Port Sanitary Authorities was addressed by W. H. Williamson, Chairman Port of London Sanitary Authority; that of the Medical Officers of Health by E. Sergeant, M.R.C.S., L.R.C.P., L.S.Sc., medical officer of health, Lancashire County Council; that of Engineers and Surveyors by Philip H. Palmer, M.Inst.C.E., borough engineer, Hastings; and that of Sanitary Inspectors by W. G. Cooper, chief sanitary inspector, Bournemouth. A paper was also read by W. Hunting, F.R.C.V.S., on "Scarlet Fever in Relation to Cow's Milk."

